# Effects of backward walking exercise using lower body positive pressure treadmill on knee symptoms and physical function in individuals with knee osteoarthritis: a protocol for RCT

**DOI:** 10.1186/s13018-023-03711-x

**Published:** 2023-04-01

**Authors:** Sattam M. Almutairi, Marzouq K. Almutairi, Mansour M. Alotaibi, Mohammed Alshehri, Aqeel M. Alenazi

**Affiliations:** 1 Department of Physical Therapy, College of Medical Rehabilitation Science, Qassim University, Buraydah, 52571 Saudi Arabia; 2 Physical Therapy Department, Faculty of Applied Medical Science, Northern Border University, Arar, Saudi Arabia; 3 Physical Therapy Department, Jazan University, Jazan, Saudi Arabia; 4 Department of Health and Rehabilitation Sciences, College of Applied Medical Sciences, Prince Sattam Bin Abdulaziz University, Al-Kharj, Saudi Arabia

**Keywords:** Osteoarthritis, Arthritis, Lower extremity, Gait, Pain, Body weight

## Abstract

**Objectives:**

The primary aim is to compare the effects of backward walking exercise to forward walking exercise on knee pain, knee functions, and thigh muscle strength in individuals with mild to moderate knee osteoarthritis using lower body positive pressure, in addition to mobility functions, balance, and self-reported health status.

**Methods:**

The study is a single blind randomized clinical trial with two independent groups. This study will enroll 26 participants with mild to moderate knee osteoarthritis. The participants will be randomized into either experimental group (backward walking exercise) or control group (forward walking exercise). Both groups will use lower body positive pressure treadmill for walking exercise. Both groups will perform regular conventional exercise and worm-up exercise before walking exercise. The treatment will be three times a week for six weeks. Walking session will be up to 30 min each session. Data collection will be collected during pre- and post- intervention including primary outcomes including numeric pain rating scale (NPRS), knee injury and osteoarthritis outcome score (KOOS), and thigh muscle strength test. The secondary outcomes include five times sit to stand test (FTSTS), 3-meter backward walk test (3MBWT), timed up and go test (TUG), four square step test (FSST), functional reach test (FRT), 10-meter walk test (10-MWT), six minute walk test (6MWT), medical outcomes study short form 12 (SF-12), patient health questionnaire -9 (PHQ-9), and rapid assessment of physical activity (RAPA). An independent t-test will be used to evaluate the effect of treatment on the outcome measures.

**Results:**

Not applicable.

**Conclusion:**

Using lower body positive pressure may have promising results against knee osteoarthritis. Moreover, walking backward exercise using lower body positive pressure might add more benefits to individuals with knee osteoarthritis and help clinicians in decision making.

*Trial registration*: This study was registered in ClinicalTrails.gov (ID: NCT05585099).

## Introduction

Osteoarthritis (OA) is a joint degenerative disease affecting more often adults aged 65 year and older [1]. Globally, the pooled prevalence on knee OA was 16% in people aged 15 and older and 22.9% in those aged 40 and older [2]. Knee OA is the most common arthritis [3] that causes disability and interferes with activities of daily life (ADLs) [4], and quality of life [5], and increases the risk of fall [6]. Several symptoms are associated with knee OA including pain, stiffness, knee muscle weakness, compromised joint integrity, poor balance, and limited knee range of motion [7, 8]. However, non-surgical interventions such as physical therapy, exercise, and manual therapy along with patient education are important and often efficacious for improving patient-related outcomes in individuals with knee OA [9, 10].

Walking exercise is an effective and recommended treatment for individuals with knee OA in minimizing pain and disability [11]. Likewise, backward walking exercise was efficacious in reducing knee OA symptoms than forward walking exercise [12]. Backward running also reduces compressive stresses at the patella-femoral joint, compared with forward running [13], which may alleviate knee pain and improve knee function. Backward walking training have been reported to increase quadriceps muscle strength in individuals with knee OA [12, 14].

Adding backward walking exercise to a traditional treatment program for three weeks improved function and decreased disability in individuals with knee OA [15]. Furthermore, six weeks of backward walking exercise resulted in a higher decline in pain and functional disability compared to forward walking exercise and control groups which receive conventional physical therapy, as well as improved quadriceps muscle strength and performance [12]. However, there was no significant difference between forward walking and backward walking exercise in all outcome measures. Furthermore, forward walking exercise and backward walking exercise were on overground which may increase the force on the knee due to the body weight loading and limit the exercise duration which was 10 min.

Lower body positive pressure (LBPP) treadmill uses air pressure chamber to unload body weight during walking or running [16]. Reducing compressive forces at knee joint by decreasing body weight while walking or running often recommended to patients with knee OA [17]. Using LBPP for walking exercise stimulated weight loss and reduced knee pain [17]. LBPP has been shown to decrease knee pain in individuals with knee OA [17]. Compared to regular treadmills [18] in forward walking exercise, potentially by reducing the compressive forces at the knee joint while walking. However, previous studies only investigate forwarding walking exercise in LBPP. The advantages of backward walking exercise using LBPP have not been investigated yet. Thus, this randomized controlled trial (RCT) aims to investigate the effects of backward walking exercise using LBPP compared to forward walking on knee pain and functions in individuals with mild to moderate knee OA. We hypothesized that backward walking exercise could provide additional benefits to knee symptoms and functions more than forward walking exercise using LBPP. A secondary aim is to examine the effects of backward training compared to forward training on mobility functions, balance, and self-reported health status in this population.

## Materials and methods

### Study design

**Fig. 1:**
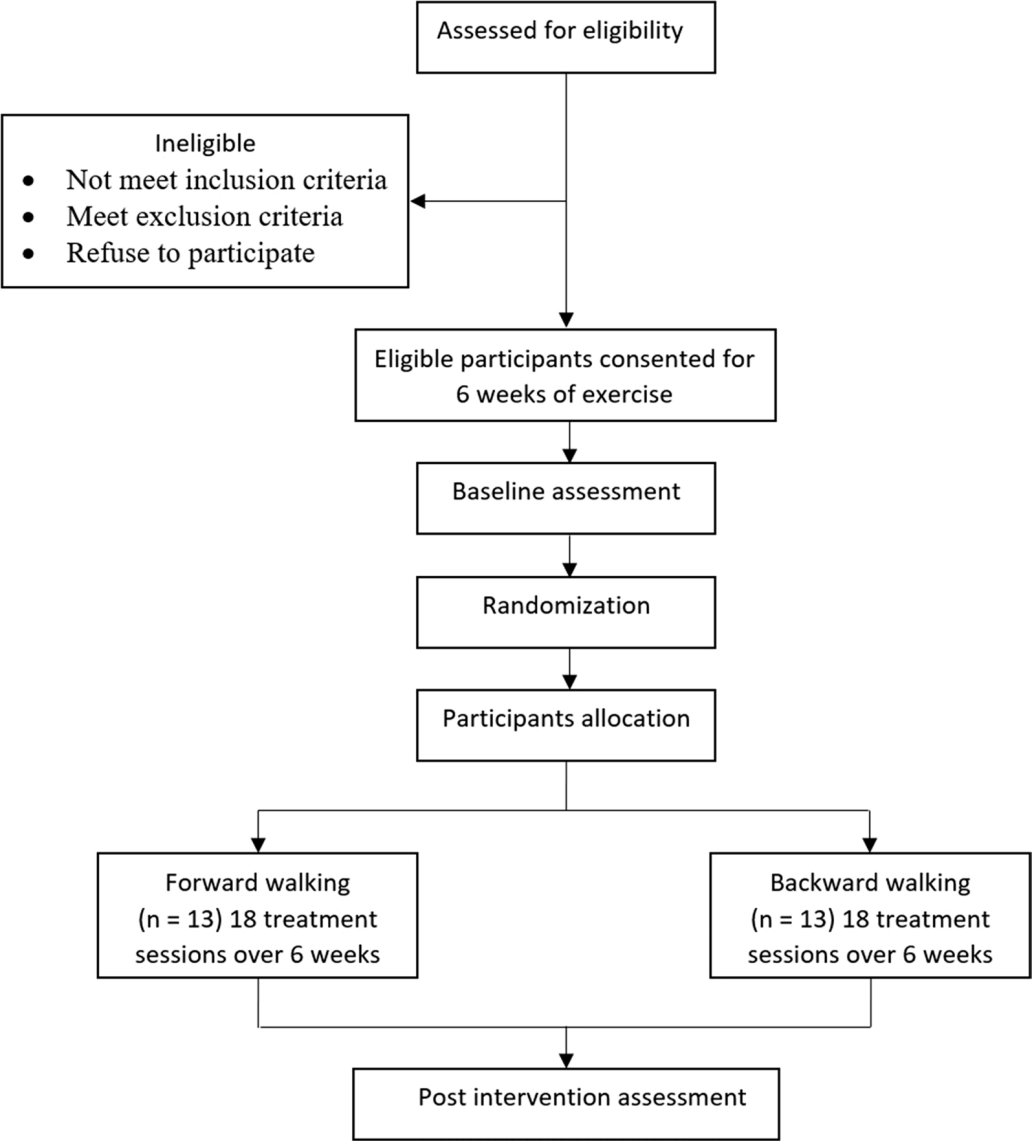
Flow diagram of study protocol

This study is a prospective two-arm single-blinded RCT. The experimental group (EG) will perform backward walking exercise, and the control group (CG) will perform forward walking exercise using LBPP device. All outcome measures will be collected at baseline (T0) and post-intervention following 6 weeks or 18th session (T1) (Fig. 1).

### Ethical approval

All procedures and intervention will be carried out in compliance with the 1964 Helsinki Declaration and its later amendments or comparable ethical standards, as well as the ethical standards of the institutional research committee. The ethical approval from National Bioethics Committee Review Boards of Qassim University was obtained before the start of the study (protocol number 22-07-07). In addition, this study was prospectively registered in ClinicalTrails.gov (18/10/2022; ID: NCT05585099).

### Participants and recruitment

**Fig. 2:**
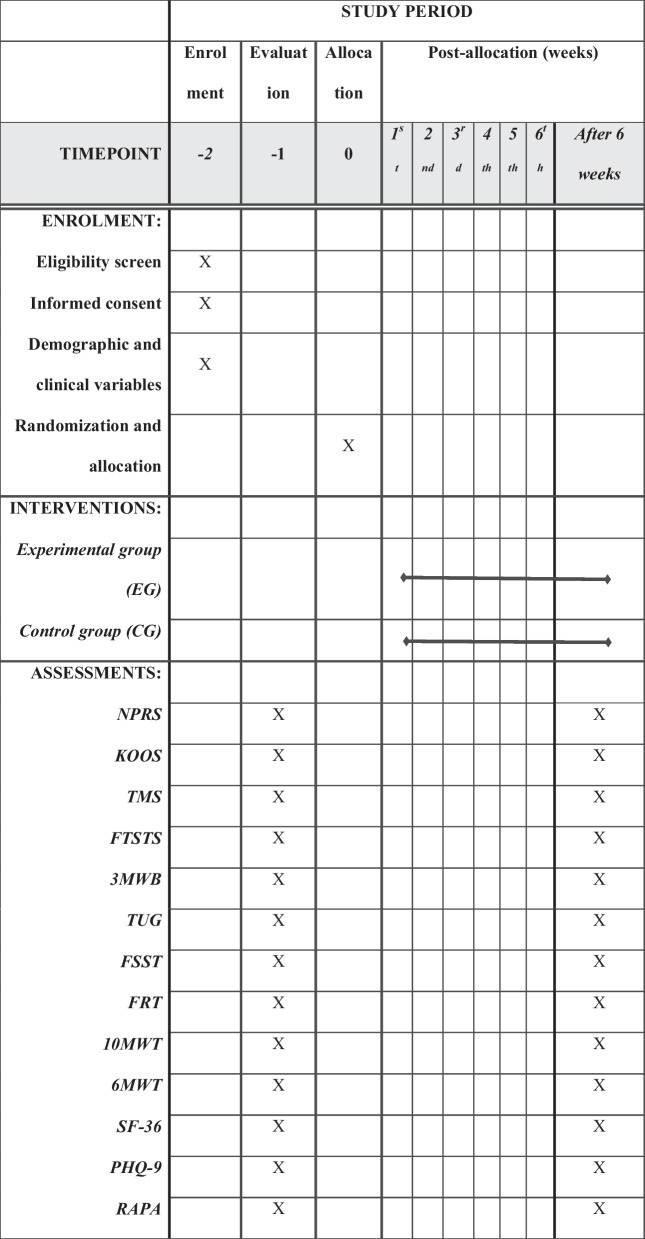
Standard protocol items: recommendations for content for the schedule of enrollment, interventions, and assessments for interventional trials (SPIRIT) checklist, (EG) experimental group, (CG) control group, (NPRS) numeric pain rating scale, (KOOS) knee injury and osteoarthritis outcome score, (TMS) thigh muscle strength test, (FTSTS) five times sit to stand test, (3MWB) 3-meter walk backward test, (TUG) timed up and go, (FSST) four square step test, (FRT) functional reach test, (10-MWT) 10-meter walk test, (6MWT) 6-minute walk test, (SF-36) short form 36, (PHQ-9) Patient Health Questionnaire-9, (RAPA) rapid assessment of physical activity

A sample consisting of 26 men and women with knee OA is required to be allocated to each group (*n* = 13 per group). The participants will be recruited from surrounding communities. All participants will read and sign an informed consent form that describes the study protocol, risks, and benefits. Figure 2 depicts the study’s expected timeline for enrollment, evaluation, and treatment. All data collection and intervention will take place at the Qassim University Medical City's physical therapy department in Saudi Arabia.

### Inclusion criteria

Participants should: (1) be ≥ 40 years old; (2) have been diagnosed with a mild to moderate knee OA by a physician and confirmed by radiograph imaging (Kellgren & Lawrence grade 1–3); (3) complain of knee pain during the past 30 days; (4) and have unilateral or bilateral knee OA. To determine knee OA diagnosis, the X-ray will be obtained from standing position using anteroposterior images. If the patient has bilateral knee OA, the most symptomatic knee indicated by the patient will be included in the outcomes.

### Exclusion criteria

Participants will be excluded if they: (1) have a history of ankle, knee, or hip injury or medical operation within the past 12 months; (2) are diagnosed with rheumatoid arthritis; (3) have used knee injection for their knee OA pain within the past year; (4) have a history of other medical conditions that would interfere with walking; (5) received physical therapy during the past 3 months for their knee OA; (6) and are pregnant.

### Outcome measures

#### Primary outcome measures procedures

##### Demographic and clinical variables

Demographic information pertaining to patient’s age, sex, and education level will be collected. Clinical variables include OA diagnosis type and severity, having other chronic conditions (e.g., diabetes mellitus and high blood pressure), prescribed medications, history of falls during the last 12 months, average pain level in the past month, body measures [height, weight, and body mass index (BMI)], and physical activity (minutes per week) will be obtained.

##### Numeric pain rating scale (NPRS)

The 11-point NPRS measures pain severity from 0 to 10, where 0 indicates "no pain" and 10 indicates "worst imaginable pain." The NPRS has excellent internal consistency (Coefficient alpha = 0.84) [19] and has been validated to evaluate pain severity [20]. Minimal clinically important difference (MCID) in individuals with osteoarthritis is from 1 to 1.7 points or a change of 15–27.9% [21, 22]. At baseline, participants will rate their pain over the past 24 h using the NPRS. The NPRS will be taken before each session, throughout gait training, and after six weeks.

##### Knee injury and osteoarthritis outcome score (KOOS)

The KOOS assesses knee pain, symptoms, and level of disability during the last week [23]. It has five subscales: Pain; Symptoms; Function in ADLs; Sports and Recreation; and Quality of Life. Each subscale has a normalized score from 0 to 100, where 0 represents extreme symptoms and 100 represents no symptoms. The test is valid, reliable, and responsive to change for people with knee OA [24]. The minimal detectable change (MDC) is from 8 to 10 points [25]. A validated Arabic version of KOOS will be utilized for this study [26].

##### Knee flexor and extensor muscles strength

An isokinetic dynamometer (Biodex Model 4Pro, N.Y. 11,967, U.S.A) will be used to assess isometric knee flexion and extension strength of both sides [27]. The hip angle will be at 85° of hip flexion and the knee angle will be at 60° of knee flexion [28]. Both legs will be tested, and the trial order will be randomized. Patients will be instructed to exert as much effort as they could for five seconds during each test. We will explain the test procedure to the participants and provide 5 min warm-up cycling before the test. To avoid fatigue, 60-s rest time between trials will be provided. The testing session will be directed by the same investigator, who will verbally encourage each participant to perform their best. The peak torque obtained over the 3 repetitions will be recorded. Peak torque measurement is considered the most valid method to assess the muscle strength in individuals with knee OA [29].

#### Secondary outcome measures procedures

##### Five times sit to stand test (FTSST)

The FTSST is an indicator of postural control, lower limb muscle strength, and balance [30]. Participants will stand up from a 45-cm chair with their arms over their chest and sit down five times as fast as they can without contacting the chair back. Start a timer at "go" and stop it when the participant's buttocks touch the chair on the fifth repeat. Participants can practice one trial before data collection. The FTSST has an excellent test–retest reliability (ICC = 0.960) in individuals with OA [31], and construct validity in people with rheumatoid arthritis [30].

##### 3-meter backward walk test (3MBWT)

The 3MBWT’s field begins and ends with marked colored tape. The participant will stand with their heels at the level of the front edge of the tape. Participants will be instructed to walk backwards 3 m when the investigator says “go” and stop when the participant passes the end-line tape. The participant can look back during the test. The test will only be administered once, and the time spend in completing the test will be recorded using a stopwatch. The investigator will walk behind the participant for safety. Participants will be advised to walk as fast as possible to the end line.

##### Timed up and go test (TUG)

The TUG evaluates an individual’s functional mobility by asking them to stand up, walk toward a cone placed of 3 m from the chair at a normal speed, turnaround 180 degrees, walk back 3 m, and sit down [32]. The TUG has an excellent reliability and validity in people with knee OA [33]. The participants will be allowed to have one-practice trials. The third TUG trial will be recorded and documented.

##### Four square step test (FSST)

The FSST is designed to assess dynamic balance and coordination. Participants will step forwards, sideways, and backwards as fast as they could over 4 canes that shaped plus sign without touching the canes. Start in square 1 facing square 2. Participants will follow the following order: square number 2, 3, 4, 1, 4, 3, 2, and 1. Participants can use walking aid during the test. Two trails will be performed, and the best trial will be documented. The trial will be repeated if the participant unsuccessfully completed the sequence. The time will be recoded when the first foot makes contact with square 2 and stopped when the last foot makes contact with square 1. The test has an excellent reliability and validity in individuals with hip OA [34].

##### Functional reach test (FRT)

Participants' stability and balance are evaluated using the FRT by measuring how far they are able to lean forward from a standing position. Participants extend their arm with a closed fist and leaning forward as possible without taking a step. On the yardstick, the beginning position will be marked at the 3rd metacarpal proximal edge. The highest achievable distance will be marked as ending position. The distance between the beginning and ending positions will be computed. One-practice trials will be permitted. The participant will complete two trials. The analysis will utilize the average of the two trials. The FRT has high intra-rater reliability (ICC = 0.98) and validity (*r* = 0.71) [35].

##### 10-meter walk test (10-MWT)

The 10-MWT evaluates walking speed over a brief period of time with or without the use of an assistive device. The participant will walk 10 m with an acceleration and deceleration zones for first and last 2 m. Only the 6 m distance in between will be used for scoring. The 10-MWT has excellent test–retest reliability with different populations [36, 37]. The 10-MWT has MCID of 0.13 m/s for significant meaningful change in older adult [37]. Participants will perform the test twice at comfortable speed and twice at fast speed, and the average of the results speeds will be utilized for the analysis separately.

##### Six minute walk test (6MWT)

6MWT measures aerobic endurance through distance walking during 6 min. Participants may take as many time as desired for standing rest; however, the investigator must continue timing. The number of standing rests and the duration of each standing will be recorded. Assistive device or braces are allowed and will be documented.

#### Medical outcomes study short form 12 (SF-12)

This medical health survey evaluates health related QoL for research and clinical practice purposes. This measure is a shortened version of medical outcomes study short form 36 (SF-36). It measures eight different aspects of health and well-being. The total score ranges from 0 to 100, where 0 reflects the worst QoL and 100 reflects the best. The Arabic version of the SF-12 has been translated and validated [38].

##### Patient health questionnaire -9 (PHQ-9)

The PHQ-9 is a questionnaire for evaluating depression symptoms. It comprises nine items, each of which asks the participants to score the severity of their depression symptoms on a 4-point Likert scale over the last two weeks ranging from 0 “Not at all” to 3 “Nearly every day”. A total score of 5–9 indicates minimal depression symptoms, 10–14 indicates moderate symptoms, 15–19 indicates substantial symptoms, and score of 20 or higher indicates severe depression [39, 40]. This tool is reliability and validity in previous investigation [41]. This tool has been translated and validated into different languages including Arabic language [42].

##### Rapid assessment of physical activity (RAPA)

The RAPA is a subjective survey that consists of nine questions that examines the physical activity of elderly. The physical activity is classified into three categories (light, moderate, and vigorous) along with textual and graphical descriptions [43]. Arabic language of RAPA measure has been translated and validated [44].

### Procedure

#### Assignment of intervention

##### Allocation sequence generation

In a ratio of 1:1, the participants will be allocated randomly into an experimental group (EG, *n* = 13) or a control group (CG, *n* = 13), using an online randomization website (https://www.graphpad.com/quickcalcs/randomize1.cfm), a research assistant who is not involved in the intervention or data collection will generate the randomization.

##### Allocation concealment

The random assignment for each participant will be placed in an anonymous envelope. Envelopes will be prepared by an independent research assistant, who will then conceal information for the evaluator investigator. Following completing the baseline evaluation, the independent research assistant will draw an envelope and notify a trained physical therapist of group assignment for each participant.

##### Blinding

This study is a single-blinded investigation. The group assignment will be concealed from the evaluator.

##### Intervention

After signing the informed consent form, participants will fill out an intake form about demographic data and clinical variables. Additionally, they will be evaluated on inclusion/exclusion criteria. Participants meeting the inclusion criteria will be recruited in the study and assessed on the primary and secondary outcome measures. For test order, participants will be randomly assigned to one of four groups. A counterbalanced approach will be used to manage the order effects of learning and fatigue associated with the administered tests.

**Fig. 3:**
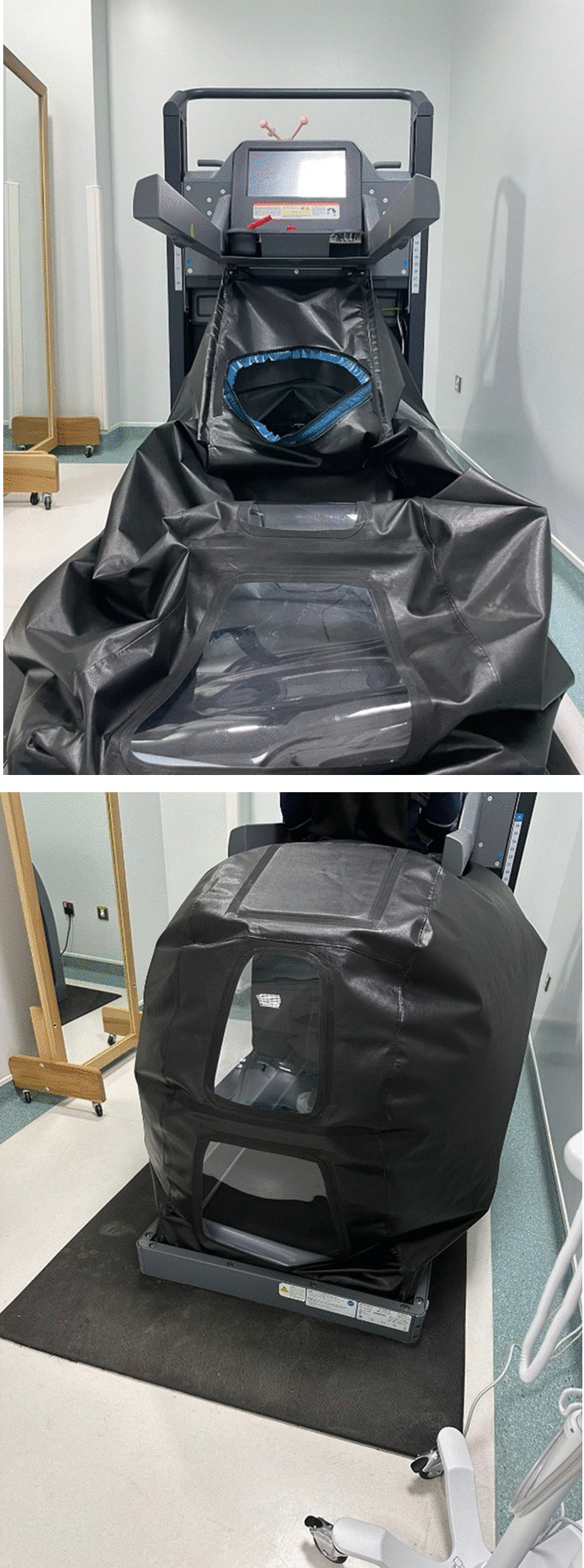
Lower body positive pressure (Alter G)

Participants will be randomized on the first day of the intervention to either a EG or CG. All participants will receive manual therapy and therapeutic exercise (Table 1) as described previously [12]. LBPP (AlterG Anti-Gravity Treadmill, Fremont, CA, USA) will be used for both forward and backward walking exercise (Fig. 3). All participants will have three sessions a week for 6 weeks (18 sessions) for up to 1 h/session. For the CG, gait training will be trained on forward walking using LBPP. The EG will be trained on backward walking using LBPP. For both groups, progression of gait training time will be instructed to gradually increase up to 30 min over the 6 weeks period. The air pressure inside chamber will be set to achieve pain free or max pain relief. Treadmill speed will be continuously assessed and increased gradually depending on the patient’s capacity. All participants will have 5 min of warm-up and cool-down exercise including heel raise, and hamstring and gastrocnemius-soleus stretches. All interventions and activities will be formed with a supervision of licensed physical therapists. Physical therapists will be trained on delivering the intervention and reporting the needed data each session. Before beginning the study, the evaluator will receive training on data collection and measuring all outcome measures.

**Table 1:** Protocol exercise for all groups

Exercise	Procedure	Frequency	Intensity	Progression
Isometric quadriceps exercise	Patients lay in a supine position. A rolled-up towel was put beneath the knee. They were instructed to maximally activate their thigh muscles in order to straighten their knee and hold the contraction for 5 s	3 days/week	1 set of 10 repetitions/twice a day	1st week: 1 set 2–3 weeks: 2 sets 3–6 weeks: 3 sets
Straight leg raising (SLR) exercise	Patients lay in a supine position. They were instructed to perform a maximum isometric quadriceps contraction prior to the lifting phase of the exercise. Then they were instructed to lift the leg up to 10 cm above the plinth and hold the contraction during the lifting phase for 10 s	3 days/week	1 set of 10 repetitions/twice a day	1st week: 1 set 2–3 weeks: 2 sets 3–6 weeks: 3 sets
Isometric hip adduction exercise	Patients lay in a supine position. A small pillow was put between the knees. They were instructed to perform isometric hip adduction exercise while pressing the pillow between the knees and to maintain the adduction with contraction for 5 s	3 days/week	1 set of 10 repetitions/twice a day	1st week: 1 set 2–3 weeks: 2 sets 3–6 weeks: 3 sets
Terminal knee extension exercise	Patients lay in a supine position. The affected knee is flexed about 30 degrees over a rolled towel. The patients were instructed to extend the knee to zero degree and hold it for 5 s then gradually flex the knee to starting position	3 days/week	1 set of 10 repetitions/twice a day	1st week: 1 set 2–3 weeks: 2 sets 3–6 weeks: 3 sets
Semi-squat exercise	Patients were asked to stand against the wall and performed semi-squat to 45- degrees flexion at knees and held this position for 30 s	3 days/week	1 set of 10 repetitions/twice a day	1st week: 1 set 2–3 weeks: 2 sets 3–6 weeks: 3 sets
Leg press exercise	Patients were asked to perform leg press exercise on a standard leg press machine. Patients were asked to press the machine to extend the knee to zero degree and hold it for 5 s then gradually flex the knee to starting position	3 days/week	1 set of 10 repetitions/twice a day	1st week: 1 set 2–3 weeks: 2 sets 3–6 weeks: 3 sets

From Alghadir et al. [12] with permission

##### Sample size calculation

G Power software analysis version 3.1.9.2 (University of Kiel, Kiel, Germany) was used to determine the sample size using pain level changes following intervention from a prior study [12]. With a small effect size of 0.29, *α* level of 0.05, and power of 0.80, the required sample size is 22 participants (11 participants per group). With a 20% attrition rate, there will be a total of 26 participants for total sample (13 participants in each group).

### Statistical analysis

The collected data will be analyzed with IBM SPSS version 28.0 (IBM Corp., Armonk, NY, USA). The descriptive demographic data (means, medians, standard deviation, and frequencies) include age, gender, onset of knee OA, height, weight, knee involved, and all primary and secondary outcome measures will be computed and compared between two groups at the baseline using independent t-test. The Shapiro–Wilk test will be used to evaluate the normality assumption. A dependent *t*-test or Wilcoxon signed rank test will be performed to evaluates the difference between pre-intervention and post-intervention. The independent *t*-test or Mann–Whitney *U*-test will be performed to evaluate the difference between groups at the baseline and post-intervention. The level of significance will be set at *p* < 0.05.

### Ethics statement and consent to participate

The studies involving human participants were reviewed and approved by the National Bioethics Committee Review Boards. The patients/participants will be provided a written informed consent to participate in this study.

### Data management

By encoding the participants' names, all participants will be informed and assured that all identifiable data will be kept confidential before, during, and after the study. Individualized participant files will be numbered and stored in a lockable cabinet and access will be restricted. The copies of the consent form will be kept in a separate cabinet due to the identifying information and restricted access will be applied.

## Discussion

The findings of this study will add meaningful contribution to the literature about the effect of backward walking exercise using a newly developed tool LBPP that adds potential beneficial effect to people with knee OA. The results will help healthcare providers such as physical therapists and researchers in managing and choosing appropriate non-pharmacological and non-surgical interventions for people with knee OA.

Lack of long-term follow-up may limit the ability to measure long-term effects, and we cannot measure it due to limited resources. Another limitation will be potentially the presence of multiple comorbidities that could interfere with participants performance in this study’s measures.

### Future directions and clinical implications

We have proposed a study protocol where knee pain in people diagnosed with OA is treated conservatively by walking exercise using LBPP. The primary goal of the study is to assess whether backward walking exercise using LBPP can alleviate knee pain symptoms in individuals diagnosed with OA. We believe that specific combinations of using a LBPP treadmill and doing backward exercises will relieve knee pain. As such, conservative treatment might be more cost-effective and have better outcomes than other treatments, such as surgical procedures.

## Data Availability

Datasets analysis will be available from the corresponding author upon reasonable request after publication of the trial findings.

## Abbreviations

OA: Osteoarthritis
ADLs: Activities of daily living
TKA: Total knee arthroplasty
LBPP: Lower body positive pressure
RCT: Randomized control trial
EG: Experimental group
CG: Control group
T0: Baseline
T1: Time after 18 sessions
BMI: Body mass index
NPRS: Numeric pain rating scale
KOOS: Knee injury and osteoarthritis outcome score
MDC: Minimal detectable change
N-m: Newton-meter
Kg: Kilogram
FTSST: Five times sit to stand test
3MBWT: 3-M backward walk test
TUG: Timed up and go test
FSST: Four square step test
FRT: Functional reach test
10-MWT: 10-Meter walk test
6MWT: Six-minute walk test
SF-12: Medical outcome study short form 12
PHQ-9: Patient health questionnaire
RAPA: Rapid assessment of physical activity
CA: California
USA: United States
